# Delayed Addition of Template Molecules Enhances the Binding Properties of Diclofenac-Imprinted Polymers

**DOI:** 10.3390/polym12051178

**Published:** 2020-05-21

**Authors:** Laura Anfossi, Simone Cavalera, Fabio Di Nardo, Giulia Spano, Cristina Giovannoli, Claudio Baggiani

**Affiliations:** Department of Chemistry, University of Torino, 10125 Torino, Italy; laura.anfossi@unito.it (L.A.); simone.cavalera@unito.it (S.C.); fabio.dinardo@unito.it (F.D.N.); giulia.spano@unito.it (G.S.); cristina.giovannoli@unito.it (C.G.)

**Keywords:** diclofenac, mefenamic acid, molecular imprinting, delayed addition, binding isotherm, binding selectivity, imprinting factor

## Abstract

It has been reported that in the molecular imprinting technique, the use of preformed oligomers instead of functional monomers increases the stability of the non-covalent interactions with the template molecule, providing a sharp gain in terms of binding properties for the resulting imprinted polymer. Based on this theory, we assumed that the delayed addition of template molecules to a polymerization mixture enhances the binding properties of the resulting polymer. To verify this hypothesis, we imprinted several mixtures of 4-vinylpyridine/ethylene dimethacrylate (1:6 mol/mol) in acetonitrile by adding diclofenac progressively later from the beginning of the polymerization process. After polymerization, the binding isotherms of imprinted and non-imprinted materials were measured in acetonitrile by partition equilibrium experiments. Binding data confirm our hypothesis, as imprinted polymers prepared by delayed addition, with delay times of 5 and 10 min, showed higher binding affinity (*K*_eq_ = 1.37 × 10^4^ L mol^−1^ and 1.80 × 10^4^ L mol^−1^) than the polymer obtained in the presence of template at the beginning (*K*_eq_ = 5.30 × 10^3^ L mol^−1^). Similarly, an increase in the imprinting factor measured vs. the non-imprinted polymer in the binding selectivity with respect to mefenamic acid was observed. We believe that the delayed addition approach could be useful in prepar imprinted polymers with higher binding affinity and increased binding selectivity in cases of difficult imprinting polymerization.

## 1. Introduction

Molecularly imprinted polymers (MIPs) are obtained by polymerizing a mixture of cross-linkers and functional monomers in the presence of a template molecule (i.e., the pre-polymerization mixture) [1,2,3]. The current molecular imprinting concept assumes that the origin of imprinted binding sites is the non-covalent complexes between the template molecules and functional monomers in the pre-polymerization mixture, and that the polymerization process stabilizes these complexes through the formation of a dense three-dimensional polymeric network around the template molecules [4,5]. Typically, the resulting MIP shows high affinity for the template molecule, with binding equilibrium constants (*K*_eq_) on the order of 10^3^–10^5^ L mol^−1^; whereas in pre-polymerization mixtures, the strength of the template–functional monomer interaction is usually much weaker, with *K*_eq_ values between 1 and 10 L mol^−1^ [6,7]. This binding enhancement is attributed to the growth of a three-dimensional polymer network that progressively embeds the template molecules. Because of the oligomeric chains’ flexibility, conformational changes maximizing the interactions with the template are allowed. The result is a net entropic gain, but while polymerization is proceeding, the newly formed cross-links rapidly stiffen the polymer structure around the template and freeze the binding pocket in its definitive shape.

The role of the oligomeric chains as functional “macromonomers” has been shown in several ways. Efficient target rebinding has been reported when linear pre-polymerized oligomeric chains that were synthesized onto the surface of silica beads were used to imprint cholic acid [8], uric acid [9], and the alkaloid cytisine [10]. In this approach, macromonomers are formed in situ before the beginning of the polymerization process, and cross-linking is achieved by the addition of a proper bifunctional reagent after the addition of the template. Porogen-soluble linear oligomers have been synthesized and directly used as functional macromonomers to imprint (–)-cinchonidine [11], theophylline [12], bisphenol A, atrazine, and ketoprofen [13]. Several other proteins have been imprinted through a similar approach in the so-called “assisted recognition by polymeric chain” (ARPC) method [14,15,16,17,18,19,20]. The main drawbacks associated with the use of pre-polymerized oligomeric chains as functional macromonomers consist of complexity in their preparation (requiring long, multistep synthetic methods), the difficulty of their isolation from the reaction mixture, and difficulty in their manipulation due to their viscous nature. A possible alternative to overcome these drawbacks could consist of avoiding the isolation of the pre-polymerized functional macromonomers, by directly using oligomeric chains formed early in situ at the beginning of the bulk polymerization process, before reaching the gelling stage.

Since diclofenac is a proof-of-concept template molecule, the goal of this paper is to show that an enhanced imprinting effect can be obtained by delaying the addition of the template molecule because of the presence of a large number of growing oligomeric chains that are able to settle multiple non-covalent interactions with the template itself. We have experimentally verified this hypothesis by studying a series of diclofenac-imprinted polymers that were prepared by adding the template molecule at fixed times after the start of polymerization and comparing their binding properties with those of a similar MIP that was prepared using the usual approach, that is, by adding the template at the start of the polymerization process.

## 2. Materials and Methods

### 2.1. Materials

2,2′-Azobis-(2-methylpropionitrile), diclofenac sodium (DIC), ethylene dimethacrylate, mefenamic acid (MEF), and 4-vinylpyridine were purchased from Sigma-Aldrich-Fluka (Milan, Italy). Polymerization inhibitors were removed from the monomer solutions by clean-up on activated alumina. Acetic acid, acetonitrile, and methanol were purchased from VWR International (Milan, Italy). All the reagents used were of analytical grade. Diclofenac was transformed in its free acid form as follows: 250 mg of sodium salt was dissolved in 40 mL of deionized water. Then, the pH was adjusted to 1 using aqueous hydrogen chloride 1 M, and the turbid suspension was extracted three times using 15 mL of chloroform. The organic fractions were dried over anhydrous sodium sulphate and evaporated to dryness under reduced pressure, yielding the free acid quantitatively. Diclofenac and mefenamic acid stock solutions were prepared by dissolving 25 mg of the substance in 25 mL of acetonitrile then stored in the dark at −20 °C.

### 2.2. Polymers Preparation

A 300 µg/mL diclofenac solution in acetonitrile was prepared by dissolving the template under sonication and maintaining it at 60 °C under a nitrogen atmosphere. In 4-mL glass vials equipped with Mininert valves, pre-polymerization mixtures were prepared by dissolving 108 µL of 4-vinylpyridine (1.0 mmol), 1.13 mL of ethylene dimethacrylate (6 mmol), and 2 mg of 2,2′-azobis-(2-methylpropionitrile) in 400 µL of acetonitrile. The vials were purged with nitrogen, sealed, and left to polymerize at 60 °C. The template was added at 0, 5, 10, 15, 20, and 30 min from the start of polymerization by adding 245 µL of diclofenac solution (0.25 mmol) with an HPLC syringe and rapidly vortexing the vials. After overnight polymerization, the obtained bulk polymers were broken with a steel spatula, mechanically ground in a mechanical ball mill, and wet-sieved to 15–38 µm particle size. The particulate was packed in 5-mL polypropylene SPE cartridges and exhaustively washed with methanol–acetic acid 9:1 (*v/v*) until the polymers were deemed free from diclofenac by HPLC analysis of the eluates. Then, the particulate was washed with abundant acetonitrile, dried in the oven at 80 °C, and stored at room temperature. A blank polymer (NIP) was prepared and treated in the same manner, but without the template.

### 2.3. Light Scattering of Polymerization Mixtures

Pre-polymerization mixtures were polymerized for 5, 10, 15, and 20 min, then diluted 1:1 (*v*/*v*) with 5 mol L^−1^ hydroquinone in acetonitrile to stop the polymerization process. The size distribution of the macromonomers was determined at 25 °C using an ALV/NIBS-HPPS particle sizer equipped with an ALV-5000 multiple tau digital correlator (ALV, Langen, Germany). The measured autocorrelation function of the scattered light was processed by ALV Correlator software (ver.3.0), obtaining the number-weighted size distribution of the macromonomers.

### 2.4. HPLC Analysis

Reverse-phase HPLC analysis was performed on the Nova-Pak C18 (125 × 3.9 mm, 5 µm) from VWR (Milano, Italy). The HPLC apparatus was a LaChrom *Elite* (L-2130 constant-flow quaternary pump, L-2400 UV-Vis detector, L-2200 autosampler, and data acquisition system EZChrom *Elite* 3.1) from VWR Hitachi (Milan, Italy). The mobile phase was a 55:45 (*v/v*) acetonitrile–0.02 mole L^−1^ sodium phosphate buffer with a pH of 4.6. The flow rate was set at 1.0 mL/min, the injection volume was 5 µL, and the detection wavelength was 278 nm.

Diclofenac and mefenamic acid standard solutions at concentrations ranging from 2 to 200 µg/mL were prepared in the mobile phase immediately before use. The standards were analyzed three times consecutively and peak areas were plotted against the concentration. The calibration plot was drawn by using a weighted linear regression (weight = 1/conc, r^2^ = 0.998). The limit of quantification (LOQ_DIC_ = 2.8 µg/mL, LOQ_MEF_ = 2.6 µg/mL) was calculated as LOQ = 10 Sy/b, where Sy is the standard error of the response and b is the slope of the calibration plot.

### 2.5. Equilibrium Batch Rebinding

About 10 mg of the polymer was exactly weighed in 2-mL flat-bottom amber glass vials. Then, 500 µL of acetonitrile solutions containing increasing amounts of diclofenac or mefenamic acid ranging from 4 to 200 µg were added and sonicated for 10 min. The vials were incubated overnight at room temperature under continuous agitation on a horizontal rocking table. The solutions were filtered on 0.22 µm nylon membranes, and free amounts of ligand were measured by HPLC analysis. Each experimental point was assessed as the average of three repeated measurements. The binding isotherms were calculated using the SigmaPlot 12 (Systat Software Inc., Richmond, CA, USA). Non-linear least-square fitting was applied to the averaged experimental data, using a simple Langmuir isotherm model [21,22]:

(1) $$B=\frac{K_{\mathrm{eq}}B_{\max}F}{1+K_{\mathrm{eq}}F}\;,$$

where *B* is the ligand bound to the polymer, *F* is the ligand not bound to the polymer, *K*_eq_ is the equilibrium binding constant, and *B*_max_ is the binding site concentration. To ensure robust results, weighted (1/y) Pearson VII limit minimization was chosen as the minimization method. To avoid being trapped in local minima, which might give incorrect results, the fitting was carried out several times by using different initial guess values for the isotherm parameters.

The imprinting factor, *IF*, was calculated as the ratio between the equilibrium binding constants relative to the imprinted and non-imprinted polymers:

(2) $$IF=\frac{K_{\mathrm{eq}}^{MIP}}{K_{\mathrm{eq}}^{NIP}}.$$

The binding selectivity, *α*, was calculated as the ratio between the equilibrium binding constants relative to mefenamic acid and diclofenac:

(3) $$\alpha =\frac{K_{\mathrm{eq}}^{MEF}}{K_{\mathrm{eq}}^{DIC}}.$$

## 3. Results and Discussion

### 3.1. Synthesis of Imprinted Polymers

A preliminary study on the gelation time of the pre-polymerization mixtures was performed in the same experimental conditions used to prepare diclofenac-imprinted polymers. They were based on a previously reported formulation producing an imprinted polymer with good binding properties towards the chosen template molecule [23]. These experiments show that turbidity was clearly appreciable with the naked eye after about 40 min from the start of the thermal polymerization process. Early formation of nanogel particles in the pre-polymerization mixture was confirmed by dynamic light scattering measurements performed 5, 10, 15, and 20 min from the start of the polymerization (Figure S1). The presence of nanoparticles became experimentally measurable from 10 min onwards and the corresponding diameters were 15.5 ± 2.9 (10 min), 18.8 ± 4.4 (15 min), and 24.5 ± 8.3 nm (20 min), with a polydispersity index (PDI) of 0.61, 0.68, and 0.82, respectively.

**Figure 1:**
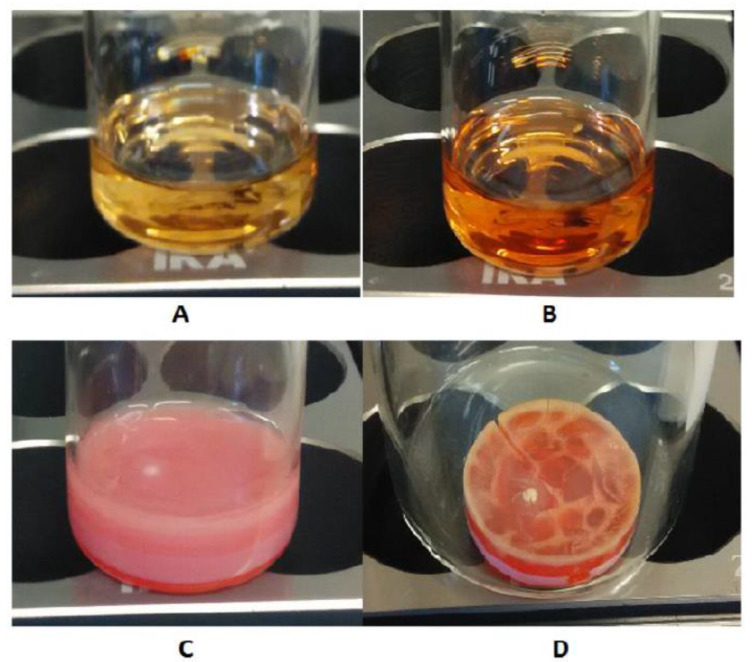
(**A**) The MIP-10 polymerization mixture immediately before the addition of diclofenac; (**B**) The MIP-10 polymerization mixture after the addition of diclofenac; (**C**) The non-imprinted polymer (NIP) polymerization mixture immediately after gelation; (**D**) The MIP-10 polymerization mixture after complete gelation. MIP-10: molecularly imprinted polymers (MIPs) prepared by template addition 10 min from the start of the polymerization.

On the basis of these preliminary studies, it was decided to carry on with the delayed addition experiments by adding preheated diclofenac solutions to the polymerization mixtures at 5, 10, 15, 20, and 30 min from the start of the polymerization process. Immediately after the addition of the template, a clear change in the solution color from yellow to dark orange was observed for all the considered mixtures. In addition, after the gelling phase started, the color of bulk polymers progressively changed from dark orange to deep red. On the other hand, the addition of acetonitrile only to the control mixture polymerized without the template did not affect the solution’s color at all, although a change from yellow to pink after the gelation of the mixture was observed (Figure 1). In fact, as previously reported in the literature [24], when 4-vinylpyridine is used as the functional monomer, the change in the adsorption spectra after template addition would qualitatively confirm the presence of non-covalent interactions between the template molecules and pre-polymerization mixtures, without giving a direct insight on the extent of such interactions. However, it is necessary to add that the color of the polymerization mixture in the presence of the template can be attributed with good approximation to the presence of complexes between the oligomers in the formation, the template itself, and traces of metal present and deriving from the alumina used to remove the inhibitor radicals from the monomers [25]. Since these complexes are known and reported in the literature [26,27], their contribution cannot be ruled out a priori.

### 3.2. Binding Properties of Imprinted Polymers

**Figure 2:**
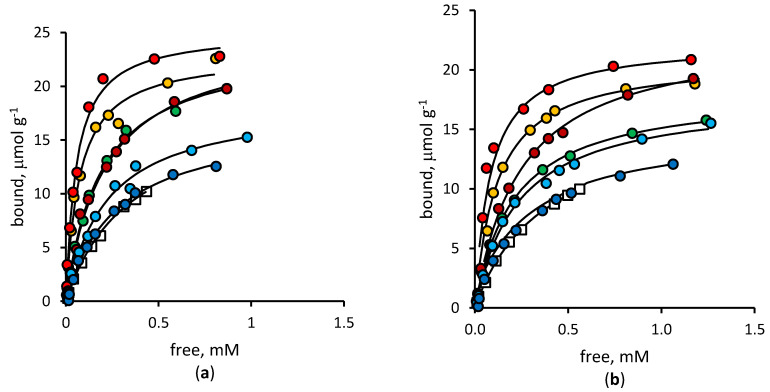
Binding isotherms for NIP (open squares), MIP-0 (green circles), MIP-5 (yellow circles), MIP-10 (red circles), MIP-15 (dark red circles), MIP-20 (cyan circles), MIP-30 (blue circles). (**a**) Diclofenac; (**b**) Mefenamic acid.

The binding properties of bulk polymers were quantitatively evaluated by measuring the binding isotherms for the template diclofenac and the related mefenamic acid (Figure 2), respectively.

Considering the binding equilibrium constants reported in Table 1 (the corresponding statistical evaluation is reported as Tables S1–S3), the imprinted polymer prepared in the presence of the template at the beginning of the polymerization process (MIP-0) showed a statistically significant (α = 0.05, *n* = 11, *t* = 3.427) affinity that was higher than that of NIP for diclofenac. This is obvious, as it must happen in the case of a successful imprinting effect. It is worth noting that this difference between MIP and NIP progressively increased in the case of MIPs prepared by template addition at 5 and 10 min from the start of the polymerization (MIP-5, MIP-10), whereas it showed a sharp decrease, eventually becoming statistically indistinguishable from NIP, when the template was added 30 min from the start (α = 0.05, *n* = 11, *t* = 1.925). The same behavior could be observed in the case of mefenamic acid, where the affinity increased from MIP-0 to MIP-10, then dropped rapidly and became indistinguishable from the NIP for MIP-30 (α = 0.05, *n* = 11, *t* = 2.052).

Regarding the binding site concentration, MIP-0 showed a statistically significant difference (*p* = 0.05, *n* = 11, *t* = 4.075) with respect to NIP in the presence of diclofenac as a ligand. This difference slightly increased from MIP-5 to MIP-10, whereupon binding site concentration values started to decrease until they became statistically indistinguishable from NIP (*p* = 0.05, *n* = 11, MIP-20, *t* = 0.247, MIP-30, *t* = 1.448). Concerning mefenamic acid as a ligand representative of diclofenac-analogous molecules, the binding site concentration showed the same trend, although the values were slightly lower. It must be noted that this difference was statistically significant only for NIP and MIP from MIP-0 to MIP-10, while it was not for the remaining polymers.

**Figure 3:**
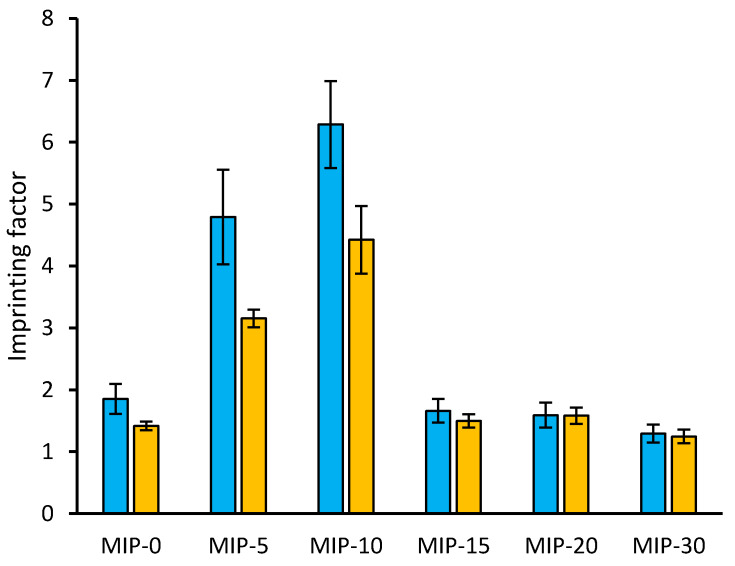
Imprinting factors (±standard error) for diclofenac (cyan bars) and mefenamic acid (yellow bars), calculated as the ratio between the equilibrium binding constants relative to the ligand for the imprinted and non-imprinted polymers.

The effect of the delayed template addition can be further highlighted by considering the imprinting factors, as reported in Figure 3. When the template was present in the polymerization mixture from the start of the process, the resulting polymer (MIP-0) showed a relatively small but statistically significant imprinting effect for both diclofenac (α = 0.05, *n* = 11, *t* = 3.509) and mefenamic acid (α = 0.05, *n* = 10, *t* = 6.003). Meanwhile, in conditions where delayed addition was employed, the imprinting effect markedly increased when the template was added after 5 and 10 min (MIP-5, MIP-10), but did not when the template was added later (MIP-15, MIP-20). Likewise, the imprinting effect was completely suppressed in the case of MIP-30.

**Figure 4:**
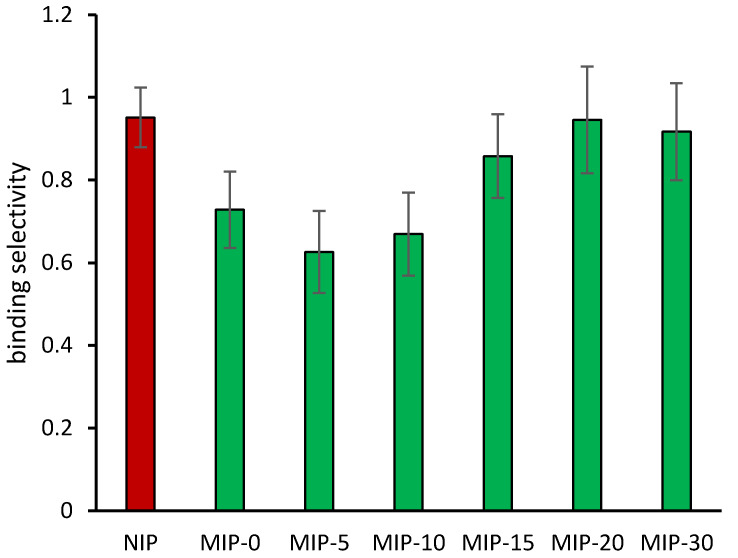
Binding selectivity (±standard error), calculated as the ratio between the equilibrium binding constants relative to mefenamic acid and diclofenac.

As a consequence of the changing binding properties of the MIP, the binding selectivity was also clearly affected by the delayed template addition in the polymerization mixture. As reported in Figure 4, the NIP did not show any binding selectivity between the template diclofenac and the related mefenamic acid (α = 0.95 ± 0.07), while the polymer prepared in the presence of the template from the beginning of the polymerization process (MIP-0) showed a moderate degree of binding selectivity (α = 0.73 ± 0.09). As in the case of the imprinting factor, in the presence of delayed addition conditions, the binding selectivity markedly increased when the template was added after 5 and 10 min (MIP-5, α = 0.63 ± 0.10; MIP-10, α = 0.67 ± 0.10), but not when the template was added 15 min from the beginning of the polymerization process (MIP-15, α = 0.86 ± 0.10). Furthermore, the binding selectivity was completely lost in the case of polymers prepared with even later addition of the template (MIP-20, α = 0.95 ± 0.13; MIP-30, α = 0.92 ± 0.12).

## 4. Discussion

From the experimental data obtained, it is worth highlighting that the addition of template molecules soon after the start of the polymerization process (5–10 min) enhanced the imprinting effect and binding selectivity, mainly by increasing the binding affinity constant of the resulting polymer. On the contrary, when template molecules were added later (15–30 min), they no longer seemed able to imprint the polymer effectively. To explain this behavior, it is useful to consider the way a bulk polymer is formed from its polymerization mixture. It is common knowledge that at the start of the polymerization process, monomers rapidly form oligomeric chains. At first, chains are poorly cross-linked and therefore highly flexible and soluble in the polymerization medium. When they grow further, cross-linking increases until they exceed their solubility limit, giving rise to nanogel particles, which may further grow and aggregate to finally form the bulk polymer. As recently demonstrated in the case of imprinted polymers prepared by solid-phase synthesis [28], the interactions between the template molecules and the pre-polymerization mixture can lead to three different outcomes:

(a) When template molecules are present in the pre-polymerization mixture from the start, they will find a reaction medium that is very rich in functional monomers, but with no oligomers. Because the polymerization process is fast and dominated by cross-linking steps, weak complexes between template molecules and functional monomers will prevail by producing an MIP with a relatively low imprinting effect.
(b) When template molecules are added after the start of the polymerization process, they will find a reaction medium enriched with functional macromonomers and only a few remaining monomers. Because the functional macromonomers are highly flexible, they will freely rearrange around the template. Chelate effects and chain desolvation greatly stabilize these complexes, producing an MIP with an enhanced imprinting effect.
(c) When template molecules are added very late in the polymerization process, they will find a reaction medium mainly composed of preformed nanogel particles that will form a continuous cross-linked macrogel. In this condition, the interactions between the template molecules and the growing polymer particles become increasingly disadvantaged, since cross-linked nanogel particles are stiffer than functional macromonomers. As a consequence, an MIP with a low or negligible imprinting effect is the more predictable outcome.

## 5. Conclusions

Our experimental data confirm that an enhanced imprinting effect occurred when a delayed addition of diclofenac as the template molecule in a polymerization mixture was performed. This behavior can be explained by the presence of growing oligomeric chains capable of settling multiple non-covalent interactions with the template itself, as well as the possible presence of metal ions. The binding isotherms highlight that the imprinting effect was due to the increase in binding affinity. We hypothesize that the experimental results described here can be related to a general behavior of any imprinted polymer prepared by radical polymerization, regardless of the nature of the template molecule, its final morphology, and the resulting structure. We speculate that this effect could be controlled by the nature of the polymerization process. Furthermore, we believe that the delayed addition approach could be useful whenever difficult imprinting polymerization takes place, as in the case of template molecules that are unable to establish strong interactions with functional monomers, and thus lead to the formation of imprinted polymers with high imprinting factor.

## Figures and Tables

**Table 1:** Calculated binding parameters (±standard error) for diclofenac and mefenamic acid measured on non-imprinted (NIP) and imprinted (MIP) polymers prepared by adding the template at 0, 5, 10, 15, 20, and 30 min from the start of polymerization.

	Diclofenac		Mefenamic Acid	
Polymer	*K*_eq_, 10^3^ L mol^−1^	*B*_max_, µmol g^−1^	*K*_eq_, 10^3^ L mol^−1^	*B*_max_, µmol g^−1^
NIP	2.86 ± 0.18	18.4 ± 0.7	2.72 ± 0.11	16.4 ± 0.3
MIP-0	5.30 ± 0.62	24.1 ± 1.1	3.86 ± 0.12	18.5 ± 0.2
MIP-5	13.70 ± 2.04	23.0 ± 1.0	8.58 ± 0.20	20.9 ± 0.1
MIP-10	17.98 ± 1.68	25.1 ± 0.7	12.03 ± 1.41	22.4 ± 1.3
MIP-15	4.75 ± 0.47	24.8 ± 1.0	4.07 ± 0.26	23.2 ± 0.6
MIP-20	4.55 ± 0.52	18.7 ± 0.3	4.30 ± 0.33	17.8 ± 0.5
MIP-30	3.70 ± 0.36	17.0 ± 0.3	3.39 ± 0.28	15.3 ± 0.5

## Supplementary Materials

The following are available online at https://www.mdpi.com/2073-4360/12/5/1178/s1. Figure S1: Dynamic light scattering of polymerization mixtures. Tables S1 and S2: Statistical evaluation of ligand binding to polymers. Table S3: Statistical evaluation of imprinting factors.
