# Supplementary material for: Delayed Addition of Template Molecules Enhances the Binding Properties of Diclofenac-Imprinted Polymers
Source: Polymers (Basel). 2020 May 21;12(5):1178. doi: 10.3390/polym12051178 (PMC7285371; doi:10.3390/polym12051178)
Supplement: Supplementary file 1 [file polymers-12-01178-s001.pdf]

polymers

MDPI

Figure S1. Dynamic light scattering of polymerization mixtures at 10 (blue line), 15 (green line) and 20 (red line) minutes

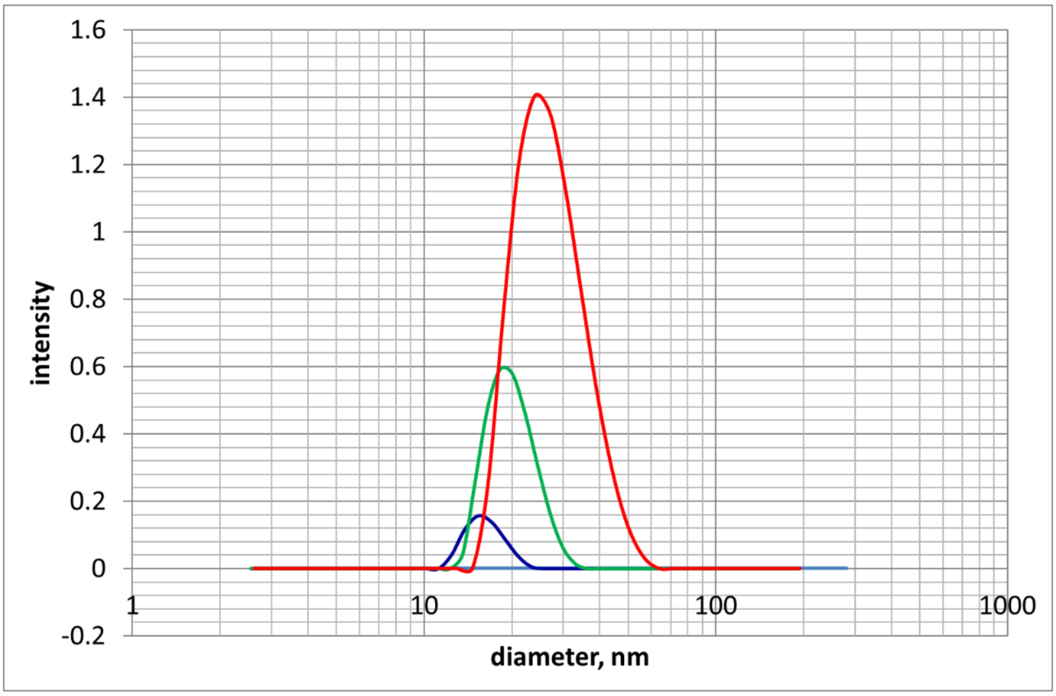

Table S1. Statistical evaluation of ligand binding to polymers. MIP were compared to NIP by calculating the Student's t-values at confidence level of 0.05. t-values denoting binding properties statistically equivalent are underlined.

	Diclofenac		Mefenamic acid	
	K_{eq}	B_{max}	K_{eq}	B_{max}
MIP-0	3.427	4.075	7.819	5.200
MIP-5	4.777	3.721	24.750	13.157
MIP-10	8.476	7.136	3.651	4.257
MIP-15	3.426	5.156	4.443	9.945
MIP-20	2.821	0.247	4.210	2.314
MIP-30	1.925	1.448	2.052	1.819

Table S2. Statistical evaluation of ligand binding to polymers. Diclofenac was compared to mefenamic acid by calculating the Student's t-values at confidence level of 0.05. t-values denoting binding properties statistically equivalent are underlined.

	K_{eq}	B_{max}
NIP	0.653	2.714
MIP-0	2.169	5.230
MIP-5	2.505	2.423
MIP-10	2.628	1.935
MIP-15	1.253	1.462
MIP-20	0.405	0.938
MIP-30	0.673	1.965

Table S3. Statistical evaluation of imprinting factors. IF for diclofenac and mefenamic acid were compared to IF=1 for NIP by calculating the Student's t-values at confidence level of 0.05. t-values denoting IFs statistically equivalent to unit (no imprinting) are underlined.

	Diclofenac	Mefenamic acid
MIP-0	3.509	6.003
MIP-5	4.955	15.902
MIP-10	7.539	6.276
MIP-15	3.448	4.529
MIP-20	2.904	4.369
MIP-30	1.991	2.190
